# Automatic counting of birds in a bird deterrence field trial

**DOI:** 10.1002/ece3.5695

**Published:** 2019-10-06

**Authors:** Elizabeth S. Simons, Mark K. Hinders

**Affiliations:** ^1^ Department of Applied Science William and Mary Williamsburg VA USA

**Keywords:** automated detection, bird detection, computer vision, object recognition, video analysis

## Abstract

1. Decreasing costs in high‐quality digital cameras, image processing, and digital storage allow researchers to generate and store massive amounts of digital imagery. The time needed to manually analyze these images will always be a limiting factor for experimental design and analysis. Implementation of computer vision algorithms for automating the detection and counting of animals reduces the manpower needed to analyze field images.
2. For this paper, we assess the ability of computer vision to detect and count birds in images from a field test that was not designed for computer vision. Using video stills from the field test and Matlab's Computer Vision Toolbox, we designed and evaluated a cascade object detection method employing Haar and Local Binary Pattern feature types.
3. Without editing the images, we found that the Haar feature can have a recall over 0.5 with an Intersection over Union threshold of 0.5. However, using this feature, 86% of the frames without birds had false‐positive bird detections. Reducing the false positives could lead to these detection methods being implemented into a fully automated system for detecting and counting birds.
4. Accurately detecting and counting birds using computer vision will reduce manpower for field experiments, both in experimental design and data analysis. Improvements in automated detection and counting will allow researchers to design extended trials without the added step of optimizing the experimental setup and/or captured images for computer vision.

## Graphical abstract

Decreasing costs in high‐quality digital cameras, image processing, and digital storage allow researchers to generate and store massive amounts of digital imagery. The time needed to manually analyze these images will always be a limiting factor for experimental design and analysis. Implementation of computer vision algorithms for automating the detection and counting of animals reduces the manpower needed to analyze field images. For this paper, we assess the ability of computer vision to detect and count birds in images from a field test that was not designed for computer vision.

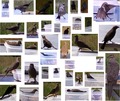

## 1 INTRODUCTION

**Figure 1:**
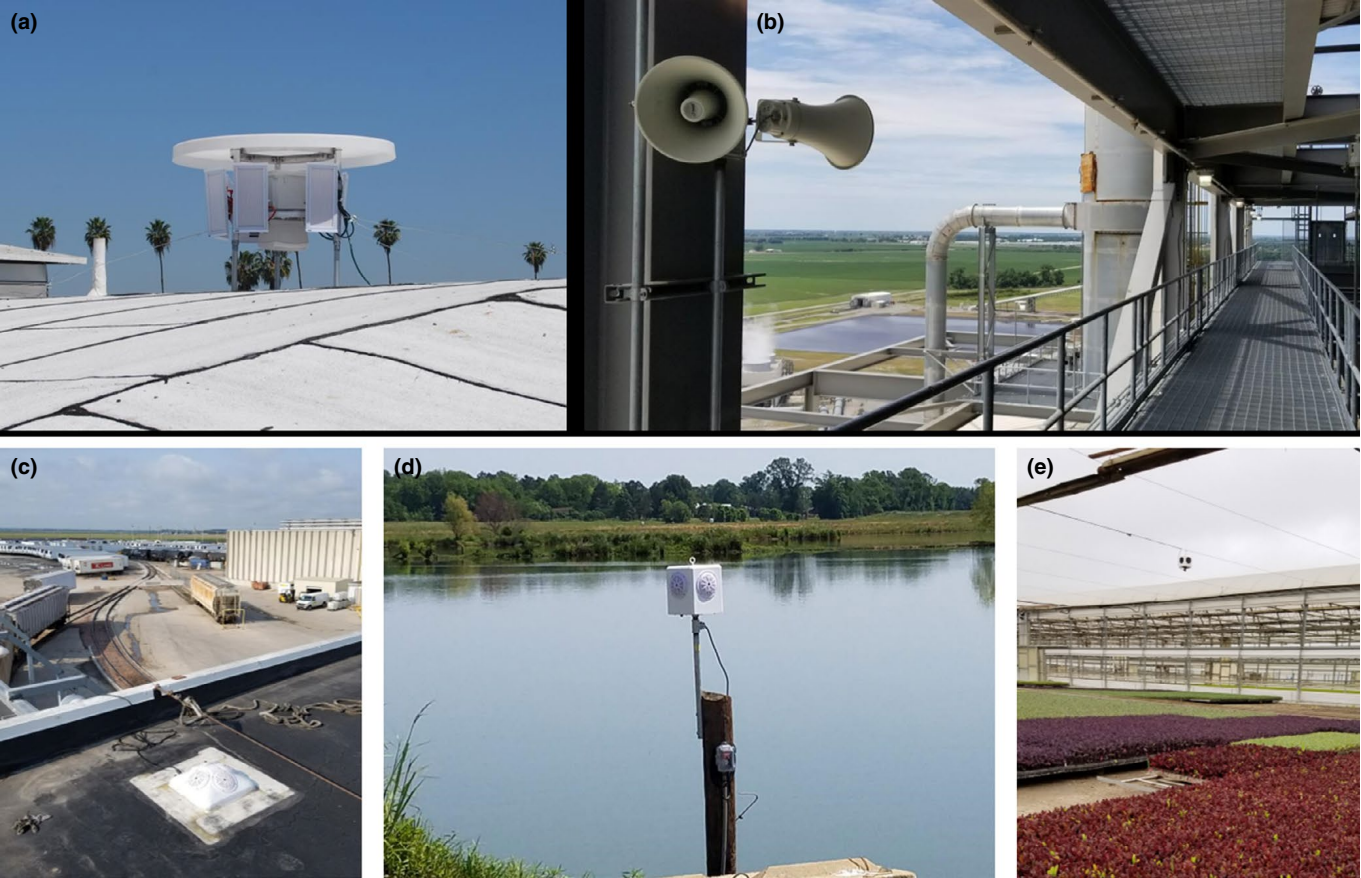
These images show some of the commercial installations of SonicNets. These installations include a directional system installed on the roof of a strip mall (a), a custom system in the superstructure of a coal power plant (b), an omnidirectional system installed on the roof of a meat processing plant (c), an ominidirectional system installed at a catfish farm (d), and an omnidirectional system installed in a plant nursery (e). Each of these systems were successful at deterring the birds from the targeted region, but there is only qualitative and anecdotal data. All images were provided by Midstream Technology

This paper explores using computer vision for automated detection and counting of birds from video captured in a field study that was not designed to use computer vision. This study was designed to determine the effect of an acoustic bird deterrent, SonicNets (http://www.sonicnets.com), in a natural environment. Previously, the effectiveness of this deterrent was determined using the time‐consuming methods of frame‐by‐frame video analysis by a researcher or point counts in the field (Mahjoub, Hinders, & Swaddle, 2015; Swaddle & Ingrassia, 2017; Swaddle, Moseley, Hinders, & Smith, 2015), or by qualitative and anecdotal evidence from customers of commercial installations of SonicNets (as shown in Figure 1, e.g.). The increased availability of inexpensive high‐quality cameras, image processing, and data storage allows for the collection of massive quantities of digital images. Automated detection techniques will reduce the cost of future field testing by reducing manpower needed to analyze the data.

Computer vision is an increasingly useful tool for environmental and biological data. Advancements in computer processing, imaging quality, and the availability of large data sets containing thousands of labeled images, such as iNaturalist (Van Horn et al., 2017), NABirds (Van Horn et al., 2015), and Caltech‐USCD Birds 200 (Wah, Branson, Welinder, Perona, & Belongie, 2011), have made it possible to create and test computer vision schemes for a variety of applications (Weinstein, 2018). Some applications include the use of satellite and digital imagery for analysis of land coverage and sediment profiles, monitoring plant phenology throughout the year, and automatic identifying and tracking of animals (Brown et al., 2016; Burton et al., 2015; Chabot & Francis, 2016; Gauci, Abela, Austad, Cassar, & Adami, 2018; O'Connell & Merryl, 2016; Romero‐Ramirez, Grémare, Desmalades, & Duchêne, 2012; Weinstein, 2018).

Computer vision techniques typically include two stages: feature extraction and classification (Wäldchen & Mäder, 2018). The feature extraction stage uses a set of training images to train an algorithm with a specific feature or series of features. There are a variety of feature types available for computer vision, often categorized into spectral, spatial, and temporal features (Bouwmans et al., 2018). After the feature extraction stage, the classification stage determines if each image in a testing set contains the object of interest. Examples of classification algorithms commonly used in biological applications are artificial neural networks, support vector machines, and cascade object detectors (Cheng & Han, 2016; Stallkamp, Schlipsing, Salmen, & Igel, 2012).

Detecting animals in their natural environment, in most cases, is trivial for humans, but the finite attention span of human investigators will always limit the amount of imagery that can be analyzed. Computer vision can reduce the amount of human analysis needed, but detecting patterns and determining the foreground of a complex image is a nontrivial task for computers. The background of fieldwork images are generally cluttered, and highly variable due to wind and lighting changes. Furthermore, the animal of interest often has little contrast to the background. To overcome these difficulties, testing conditions are often constrained. Examples include limiting the types of images used for training, focusing on species with high contrast from the background, or imposing a background that reduces clutter in the image. A common way of constraining the data is by using images where the animal comprises the majority of the pixels. This is common for studies that use training data sets to develop species identification techniques. The high‐quality labeled images that allow for detection of fine‐grain details between and within species are often zoomed in such that there is little background in the image (Berg et al., 2014). Other studies have selected species of interest because they have high contrast to the background, for example, Snowy Egrets and White Pelicans against dark backgrounds (Bohn, Möhringer, Kőrösi, & Hovestadt, 2015; Huang, Boom, & Fisher, 2015; Nadimpalli, Price, Hall, & Bomma, 2006). Another way to make computer vision more effective is by decreasing the possible orientations of the animals, often accomplished by imaging the animals from above (Abd‐Elrahman, Pearlstine, & Percival, 2005; Chabot & Francis, 2016; Jalil, Smith, & Green, 2018; Mammeri, Zhou, & Boukerche, 2016; Marti‐Puig et al., 2018; Pérez‐Escudero, Vicente‐Page, Hinz, Arganda, & Polavieja, 2014; Stern, Zhu, He, & Yang, 2015). Constraining the images in these ways can be extremely helpful for computer vision, but limits the range of possible experimental designs for understanding animal behavior.

Optimizing computer automated detection for real‐world conditions will reduce manpower needed for data analysis in a wide variety of useful experiments. To demonstrate this possibility, we trained a computer automated detection method to detect and count birds in video frames from a field test intended to be analyzed by humans. We used a cascade object detector based on the Viola‐‐Jones algorithm, developed to detect human faces (Viola & Jones, 2001). We gathered over three million images and analyzed approximately twenty thousand. Of the images analyzed, birds were present in only 2,555 frames. While there are many computer vision techniques available, we wanted to determine the effectiveness of this algorithm for this application due to its speed and robustness. Implementing an accurate bird detection algorithm will allow real‐time automated bird counting to be incorporated in a wide range of future projects, saving time, and manpower.

## 2 MATERIALS AND METHODS

### 2.1 Field trial design

**Figure 2:**
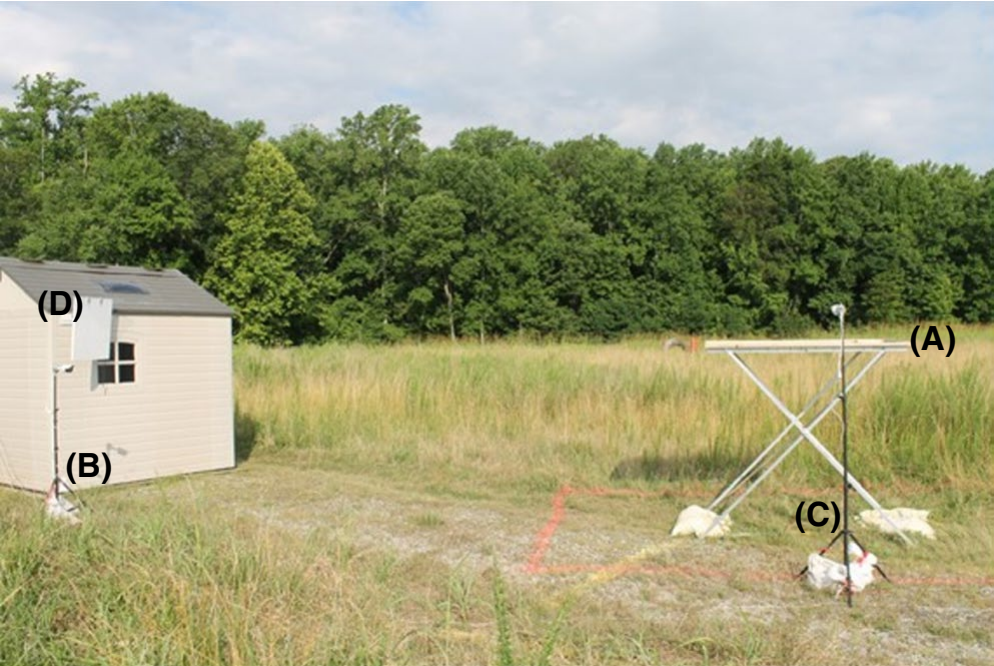
This image shows the data capture setup for this field experiment on one side of the experimental area. This setup is mirrored on the other side of the shed. Two CCTV cameras are pointed at the food table (A), one close to the shed to record the entire food table and surrounding area (B) and the other to record the top of the food table (C). The white panel mounted on the shed (D) is the parametric array speaker that allows the sound beam to cover one food table while being inaudible on the other food table

For this study, we used video stills from a field experiment conducted to replicate an aviary experiment in an open environment (Mahjoub et al., 2015). We used an acoustic bird deterrent that leverages the understanding of avian communication to design noise fields which make auditory stream segregation difficult for birds (Dent, Martin, Flaherty, & Neilans, 2016). This encourages the birds to relocate without hazing or harming the animal. The field test ran for 33 days. Two days were removed from the automatic detection testing set because there were multiple hours of video files missing from the raw data. Figure 2 shows the setup for the data capture on one side of the test area. This setup was mirrored on the other side of the shed. Two food tables (A) were placed in the field such that a highly directional parametric acoustic array speaker (D) would cover one food table with the 2–10 kHz colored noise signal of the sound beam, while the other food table remained untreated. The noise signal was applied using a 24in Audio Spotlight (https://holosonics.com/home/11-audio-spotlight-24i.html) from Holosonics (Gan, Yang, & Kamakura, 2012). A four camera Lorec HD CCTV security system (https://www.lorextechnology.com/hd-dvr-security-system/security-nvr-with-ip-cameras/LNR200-Series-B-1-p) from FLIR recorded the food tables. Each food table had one camera focused on the top of the food table (C) and another covering the food table and surrounding area (B). This experimental setup ensured the food tables were independent, while allowing for a direct comparison between treatments. The Lorec HD CCTV was programmed to record the food tables from just before 9 a.m. until just after 5 p.m. every day at a rate of one frame per second. The only human interaction with the testing site occurred before 9 a.m. and after 5 p.m. each day, when the speakers and baited birdseed containers were setup or taken down for the day.

This field test resulted in over 3,800,000 similar images, intended to be analyzed manually to determine the presence of birds. To create the testing set of images, we captured the video frame once per minute, then clipped the videos to include 357 min from 9:53 a.m. to 3:51 p.m. These were the times when no humans were present at the field test site, and all the equipment was functioning correctly. This resulted in 714 images from each day of the testing for a total of 22,134 video frames analyzed.

**Figure 3:**
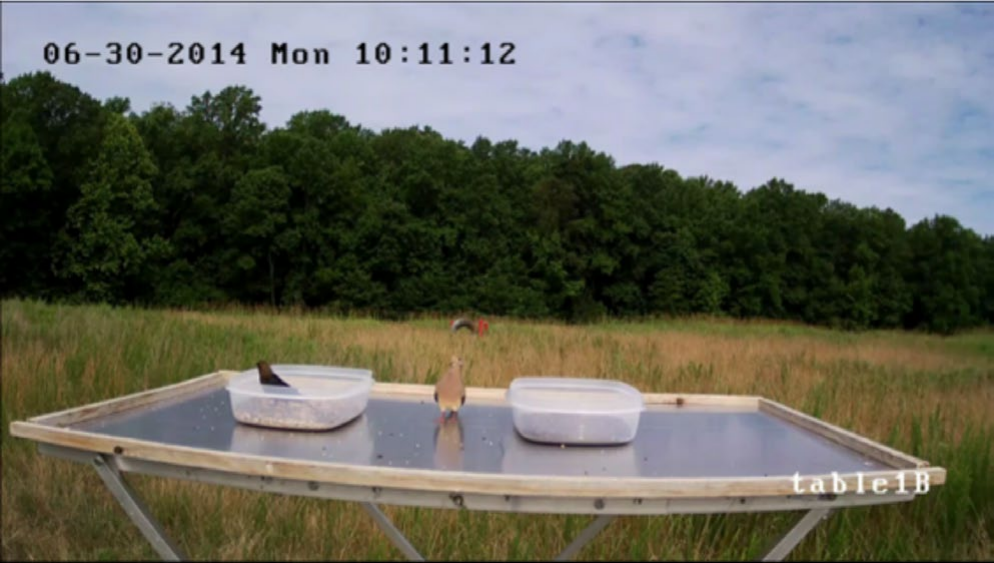
This image shows a frame of the video collected by the camera positioned to record the top of table1B, with two birds present. Here many of the characteristics that increase the complexity for the feature detection algorithm can be seen. These include the small size of the birds in the frame, two of the many possible orientations of the birds, reflections on the food table, shadows cast by the birdseed containers, obstruction of the bird by the birdseed container, and the Mourning Dove blending into the background

The automated detection algorithms were trained using video stills from the cameras focused on the tops of the food tables. The setup of this system is similar to that of a baited camera trap. There is little variation in the background from image to image, and the birds can move freely in and out of frame. In contrast to a camera trap, we recorded video continuously instead of when an animal triggered the camera. This resulted in thousands of images without birds. For our analysis, we look at table1B and table2B independently. This allows us to account for any difference in the field of view of the cameras. Figure 3 shows a still from the video file from table1B with two birds present. This figure shows some of the characteristics that increased the complexity for computer vision in this application. Since the camera placement was not optimized for computer vision, the birds only take up a small area of the frame, have a variety of orientations, and sometimes blend into the background.

Introducing the food tables into the images reduced some of the background clutter, but also introduced some complexity into the images in the form of reflections and shadows. The reflective nature of the aluminum food tables frequently created clear reflections of similar shape, size, and color saturation to the real bird. The center of Figure 3 shows such a reflection created by a Mourning Dove. In addition to reflections, the birds and birdseed containers often create shadows on the food tables. Most of the birds that visited the food tables were Brown‐headed Cowbirds, like the one seen inside the birdseed container in Figure 3. These birds have high contrast to the food table and background of the image, but have similar color saturation and tone to the shadows.

**Figure 4:**
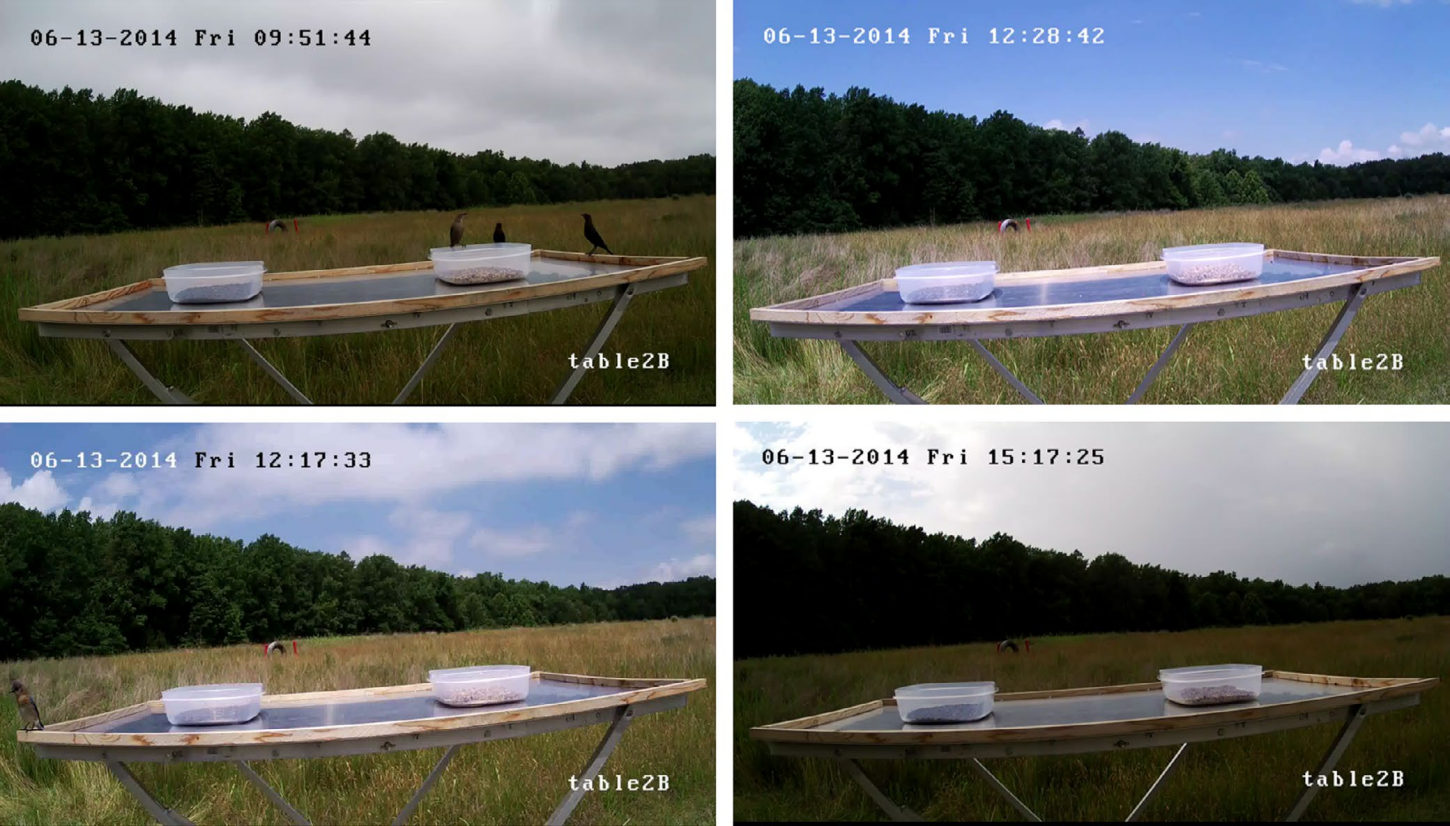
These images show some of the lighting variations that occurred in one day of testing. Changes in lighting added complexity for computer vision due to shadows and reflections on the food tables

The birds we were interested in for this application are diurnal so imaging was only necessary during daylight hours. Although we imaged during daylight hours the weather can change dramatically throughout the day in the summer in Virginia, creating significant lighting changes. Figure 4 shows four frames taken from one day of testing, showing some of the lighting variations encountered in the images. Lighting changes introduced variations of the shadows and reflections from image to image, increasing the complexity for computer vision and reduced the effectiveness of certain algorithms such as background subtraction.

**Figure 5:**
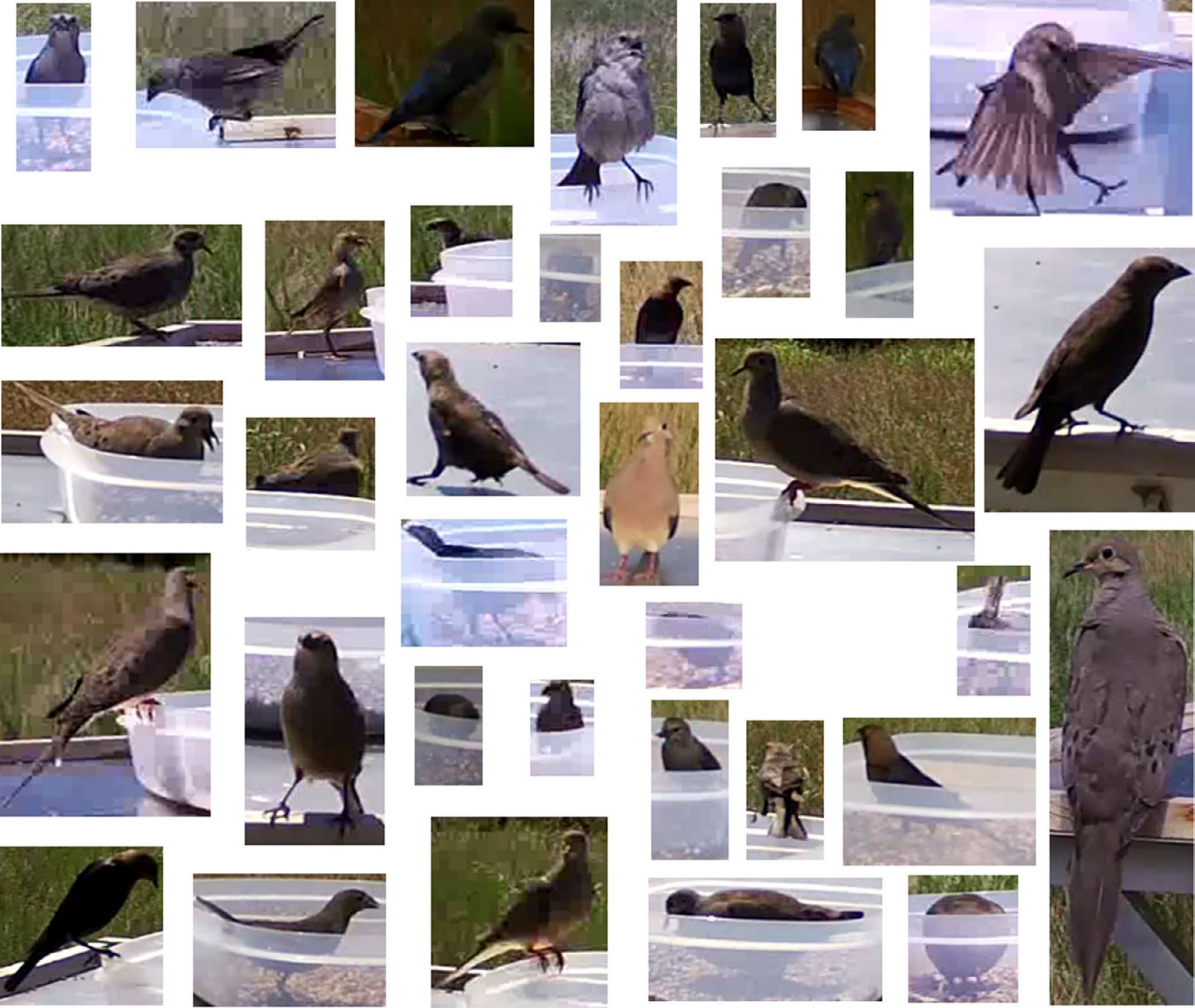
This figure demonstrates some of the complexity created by the positioning of the camera for this field experiment. Each image was created by cropping the full frame to just include the area around the bird, and the birds were not resized. These images show the inconsistent orientation of the birds. They also show that the number of pixels needed to show the entire bird varied frame to frame and bird to bird. The images also show that the birdseed containers often obscure parts of the birds

The low camera angle to the food tables increased the visible reflections and shadows on the food tables and allowed the birds to have far more possible orientations than imaging from directly above. Figure 5 shows some of the possible orientations captured in the video frames. The images of the birds were created by cropping the full sized video stills to include just the area surrounding the bird. Each video still was the same size, and the size of the bird was not changed from the original frame. This figure shows that the positioning and orientation of each bird varies significantly from frame to frame. This figure also shows that the number of pixels that contain a bird varies from image to image, making it so that the size of the bird cannot be programmed into the detection algorithm. This figure also displays a common source of false‐positive detections. When the bird is partially obscured by the birdseed container, the detection algorithm detects only the portion of the bird outside of the container.

Many of these complexities could have been avoided if the experiment had been designed to use computer vision. Even so, the images have some visual advantages compared to other studies including daylight imaging, a consistent background, imaging mostly parallel to the ground, and reduction in background clutter from the food tables. However, these are not sufficient to overcome the disadvantages, and do not make computer automated detection of birds in the images a trivial task.

### 2.2 Automated counting design

To detect and count the birds in the images, we used the Matlab Computer Vision Toolbox cascade object detector. This detector uses the Viola‐‐Jones algorithm, which has been shown to be faster and require fewer training images than other cascade object detectors (Reese, Zheng, & Elmaghraby, 2012). We chose this algorithm over a deep learning technique such as Faster R‐CNN or YOLO due to the small number of positive images in our data set. Training a neural network requires significantly more positive training images than a cascade object detector (Bowley, Andes, Ellis‐Felege, & Desell, 2017). We determined that to have enough positive images in our training set to train a neural network, the testing set would be too small. Another computer vision detection method that could be implemented for this application is background subtraction. However, we decided that the numerous background changes that occurred throughout the trial including camera positioning, lighting variation, birdseed container placement, movement due to wind and rain, and reflections on the food table would have reduced the effectiveness of this method. We also believe that the speed of the Viola‐‐Jones algorithm will allow for real‐time bird detection in future projects.

We first determined the testing set of images from the video files. Most of the images analyzed did not contain birds. Of the 22,134 video frames analyzed in our testing set only 2,555, 11%, had at least one bird present. Table 1 shows the number of frames total, with birds, and without birds for each food table. The majority of the frames containing birds were on table1B, showing that the birds preferred this food table. The data in the training set are skewed heavily toward images without birds, just like the testing set. We trained the object detector by identifying regions of interest using the Matlab Training Image Labeler application. Using frames not in the testing set, we created a set of positive training images by highlighting the birds in the video frames. A set of negative samples was created using stills from the video files without birds present. Over 2,000 total images were used to train our detector. This set of training images was then used to train the cascade object detector to detect the birds in the feature extraction stage of the algorithm.

**Table 1:** This table shows the total number of frames, the total number of frames with birds, and the total number of frames without birds for each food table

	Total frames	Frames with birds	Frames without birds
table1B	11,067	1,825	9,242
table2B	11,067	730	10,337
Total	22,134	2,555	19,579

Matlab has three built‐in detection feature types: histogram of oriented gradients (HOG), Haar features (Haar), and local binary patterns (LBP). The HOG feature converts images into intensity gradients or edge directions. The gradients are then compared between regions of the images. This feature works well for images where the orientation of the object is the same in each image (Dalal & Triggs, 2005). The Haar feature compares color intensity between rectangular regions in the images. The sum of the pixels in one region is subtracted from the sum of the pixels in the next region. This type of feature gets more complex in each stage by using smaller regions and comparing different orientations and numbers of rectangles (Viola & Jones, 2001). The LBP feature converts the images into a histogram of gray scale values. This is done by comparing a central pixel in the region to surrounding pixels. Each region is then either assigned a 0 or 1 depending on the difference between the center pixel and the surrounding pixels. This method is often used in identifying textures in images (Ojala, Pietikainen, & Maenpaa, 2002).

Using each of the three features (HOG, Haar, LBP), the cascade object detector employed machine learning to train the algorithm for detecting birds. We set the number of training stages to 20 and allowable false‐positive rate to 0.2. The training function then computed the number of positive and negative samples needed and used them in the first stage of detection. In each stage, the image is divided into regions, and bird presence is determined in each region using the feature type specified. If no bird is detected in the region, it is not considered further. If there is a region that the computer indicates could have a bird, then this region remains. The remaining regions are then divided into smaller regions and the process repeats itself. Each stage uses more complex classifiers until the maximum number of stages is met.

Once the object detector was trained, we used it to identify birds in the testing set of images. We wrote a program to loop through the video files frame‐by‐frame, automatically record the bounding boxes of the bird detections, and count the number of birds detected in each frame. This was then compared to the ground truth bounding boxes created by an investigator for the same images. We found that the Haar and LBP features worked better than the HOG feature for this application. The Haar and LBP features rely on contrast between the object and the background of the images, while the HOG method uses the shape of the objects. The effectiveness of the HOG feature was reduced because the birds are small relative to the image size and are inconsistent in position and orientation. In the initial stages of comparing the features, the HOG technique resulted in zero true‐positive detections. This led us to focus on the comparison between the LBP and Haar feature detectors for our automated bird counts.

## 3 RESULTS

**Figure 6:**
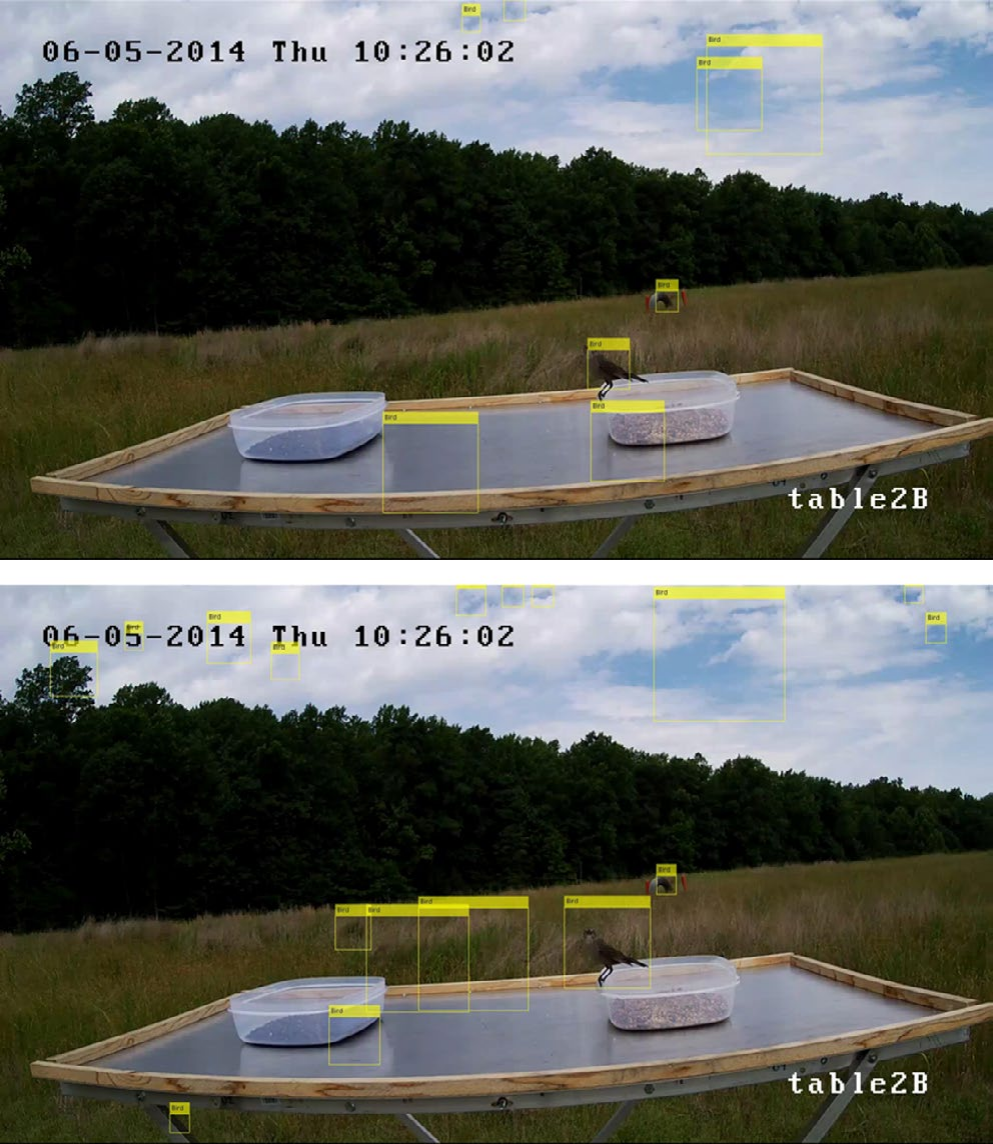
Images of the same video frame captured by the camera pointed directly at table2B. The yellow rectangles labeled “Bird” in each of the images show the bounding boxes where birds were detected using the object detection algorithm. The top image used the LBP feature detector, and the bottom image used the Haar feature detector. In both of these images it appears the automated bird detection method correctly identified the bird, but using an IoU threshold of 0.5 only the Haar feature detector produced a true‐positive detection. These images also show how weather and lighting affect the bird detection. The cloud coverage resulted in many false‐positive detections using both features

For this paper, we wanted to determine the feasibility of using computer vision to detect birds in an image from an experiment that was designed to be analyzed by humans. Using a cascade object detector trained separately with a Haar feature and LBP feature, we first wanted to determine its ability to detect birds without editing the images. Figure 6 shows the same frame of table2B with one bird present. The top image is the detection using the LBP feature and the bottom using the Haar feature. The yellow rectangles labeled “Bird” are the bounding boxes where birds were detected. In both of these images, the object detector appears to correctly identify the bird, while also flagging areas without birds.

To determine the success of our model, we calculated the metrics of precision, recall, F‐Measure, false‐negative rate (FNR), and false alarm rate (FAR) for each detector and food table with an Intersection over Union (IoU) threshold of 0.5, seen in Table 2 (Godil, Bostelman, Shackleford, Hong, & Shneier, 2014). IoU compares the ground truth bounding box and the bounding box detected by the model.

(1) $$\text{IoU}=\frac{\text{Area of Overlap}}{\text{Area of Union}}.$$

**Table 2:** This table shows precision, recall, F‐Measure, false‐negative rate, and false alarm rate for each feature detector, food table and image size with an IoU threshold of 0.5

Detector	Precision	Recall	F‐Measure	FNR	FAR
table1B full image using Haar	0.010	0.542	0.020	0.552	0.989
table1B full image using LBP	0.045	0.457	0.083	0.545	0.954
table2B full image using Haar	0.003	0.411	0.006	0.603	0.997
table2B full image using LBP	0.012	0.290	0.023	0.711	0.987
table1B cropped image using Haar	0.016	0.531	0.031	0.563	0.984
table1B cropped image using LBP	0.068	0.448	0.119	0.492	0.931
table2B cropped image using Haar	0.007	0.388	0.013	0.624	0.993
table2B cropped image using LBP	0.022	0.276	0.040	0.717	0.978

**Figure 7:**
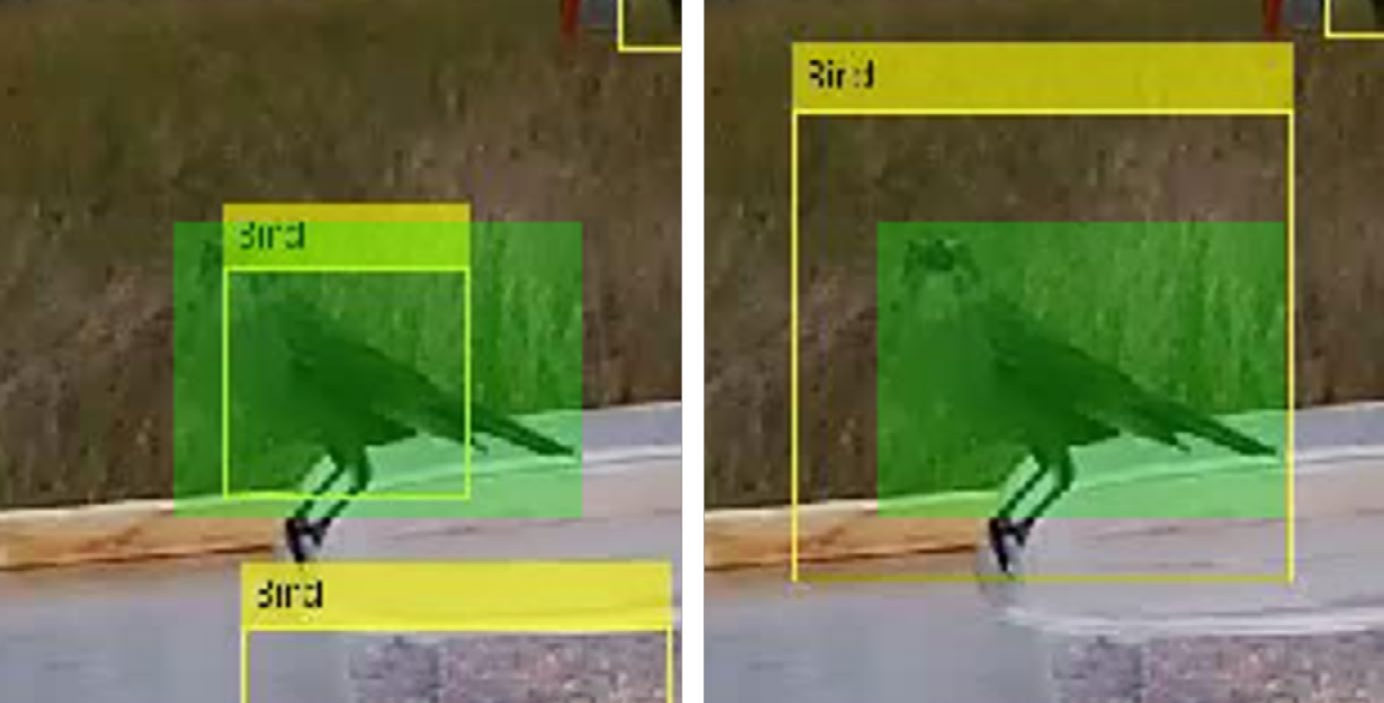
Zoomed in image of the bird seen in Figure 6. The yellow rectangles labeled “Bird” show the bounding boxes where birds were detected using the object detection algorithm and the green rectangles show the human identified ground truth. The image on the left used the LBP feature detector and the right used the Haar feature detector. The bounding box identified by the LBP feature detector captures just the body of the bird, excluding the tail, legs, and head. This results in an IoU of 0.46, below the IoU threshold of 0.5. The Haar feature detector surrounds the entire bird, resulting in an IoU of 0.52

Figure 7 shows the bird on table2B, seen in Figure 6, zoomed in to include just the area surrounding the bird. The left image used the LBP feature and the right the Haar feature for detecting the bird. The yellow rectangles labeled “Bird” in the images are the bounding boxes for the bird detected using the object detector. The light green rectangle is the human identified ground truth bounding box used for this bird. For the calculations in Table 2, a true positive is any detector bounding box that has an IoU value over 0.5 compared to a ground truth bounding box. False positives are detector bounding boxes that have IoU values under 0.5. False negatives are ground truths with no detector bounding box that has an IoU value over 0.5. True negatives are areas where neither the ground truth nor the model detected a bird.

The metrics in Table 2 are often used to evaluate computer vision models. Precision is calculated using Equation 2 and shows the percentage of accurate identifications.

(2) $$\text{Precision}=\frac{\text{True Positives}}{\text{True Positives}+\text{False Positives}}$$

In each case, the precision value for the full images was very low. The highest precision on the full images is 0.016 using the LBP feature detector on table1B. The precision for the LBP feature detector was 0.012 for table2B. The Haar feature detector had slightly lower precision of 0.01 for table1B and 0.003 for table2B. The recall for these detectors is much higher than the precision, between 0.29 and 0.54. Recall is a measure of how well the ground truths are identified, calculated using

(3) $$\text{Recall}=\frac{\text{True Positives}}{\text{True Positives}+\text{False Negatives}}.$$

The recall values seen in Table 2 show that the object detectors correctly detect each bird in the frame about half the time. The F‐Measure is calculated using Equation 4 and is an estimate of the accuracy of the system.

(4) $$\text{F-Measure}=2\times \frac{\text{Precision}\times \text{Recall}}{\text{Precision}+\text{Recall}}$$

Table 2 shows that the highest F‐Measure using the full image was 0.083 for the LBP feature detector on table1B. We also calculated the FNR using Equation 5. This is a metric of how likely a bird will be missed considering the total identifications.

(5) $$\text{FNR}=\frac{\text{False Negatives}}{\text{True Positives}+\text{False Negatives}}$$

The FAR is the number of false positives relative to the total identifications by the model as

(6) $$\text{FAR}=\frac{\text{False Positives}}{\text{True Positives}+\text{False Positives}}.$$

The FAR is a metric of how likely it is that a detection by the model is a false positive. Table 2 shows that the FAR for each detection method is very high, demonstrating the high number of false positives. This high number of false positive detections is also why the precision and F‐Measure values were so low.

**Figure 8:**
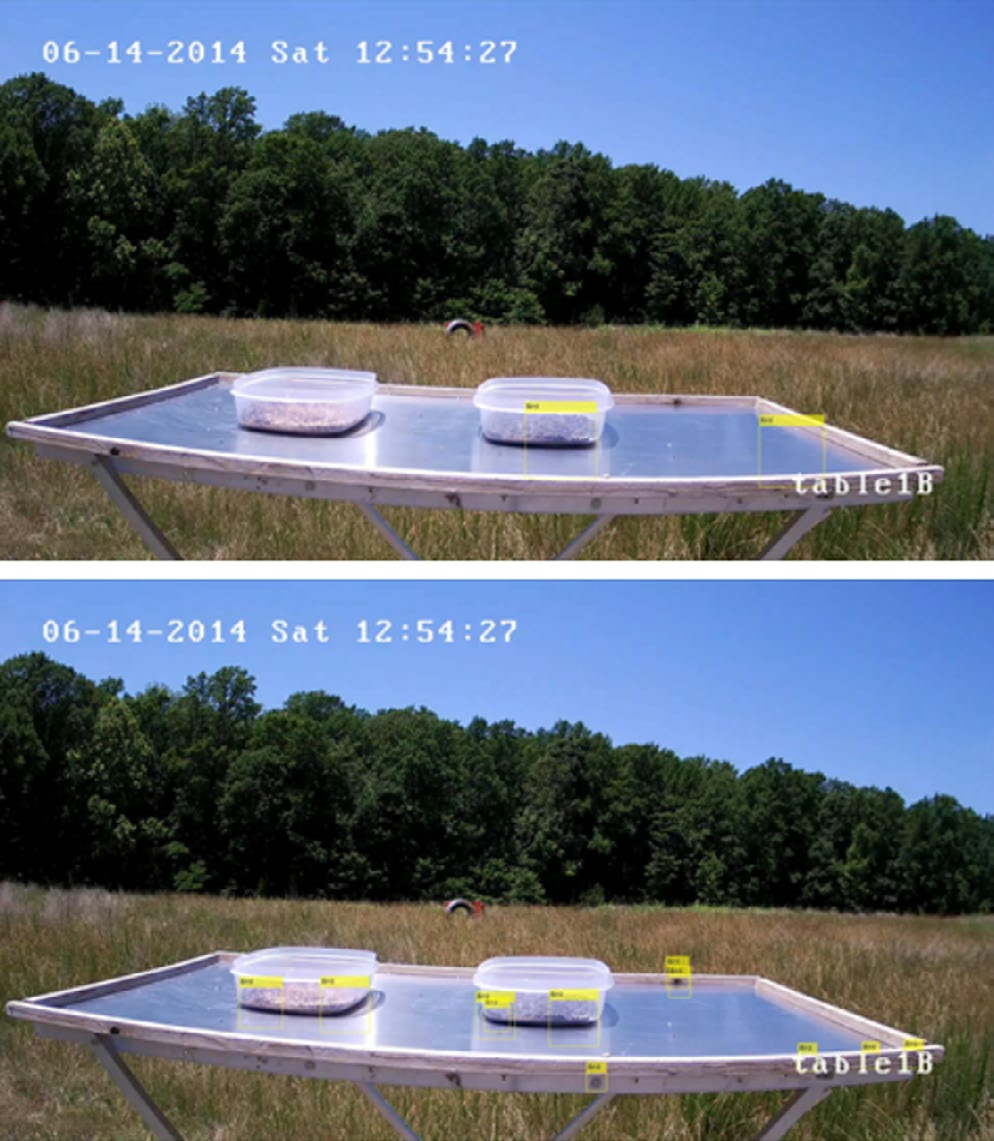
Images of the same video frame from the camera pointed directly at table1B with no birds in the frame. The yellow rectangles labeled “Bird” in each image show the bounding boxes where birds were detected using the object detection algorithm. The top image used the LBP feature detector, and the bottom image used the Haar feature detector. It can be seen that both feature detection methods had false positives. In contrast to Figure 6, these false‐positive detections occurred on the food table and were mostly due to reflections and shadows. This shows that lighting changes effect the precision of the object detector

**Figure 9:**
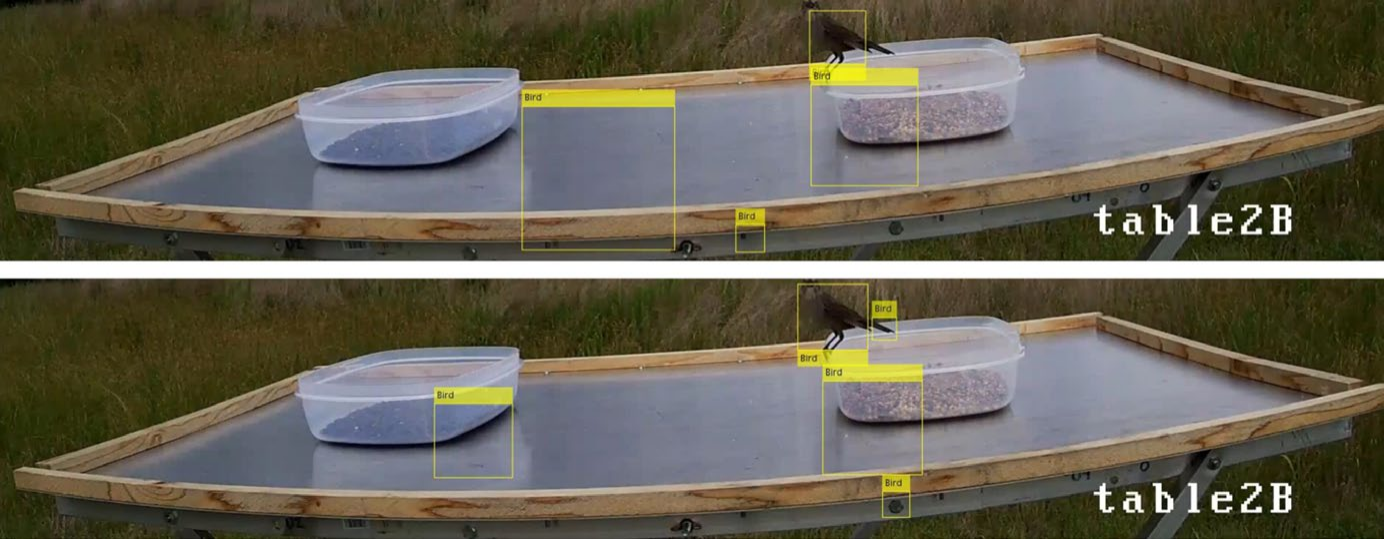
These are the images created by cropping the images in Figure 6 to include just the area surrounding table2B. The yellow rectangles labeled “Bird” in each of the images show the bounding boxes where birds were detected using the object detector algorithm. The top image used the LBP feature detector, and the bottom image used the Haar feature detector. Cropping these images reduced the total number of false positives in the frame by removing the text and clouds from the top of the image that were detected as birds in the full image. Both of these images appear to show positive detections of the bird, but the IoU in each case is <0.5

There are many factors that increased the number of false‐positive detections. Some factors include the skew in the data toward having no birds in the frame, lighting changes, shadows, and reflections. Figure 6 has more false positives than average due to the cloud coverage in that image. In comparison, Figure 8 shows a clear day with no birds present. The top image shows the bird detection using the LBP feature detector and the bottom using the Haar feature detector. The yellow rectangles labeled “Bird” are the bounding boxes where birds were detected. The number of false positives per frame is lower in Figure 8, but there are still false‐positive detections. Table 3 shows the number and percent of frames where false‐positive bird detection occurred when there were no birds on the food table. Often the false‐positive detections occurred in the background of the image, such as the bounding boxes in the clouds seen in Figure 6. To reduce these false positives, we cropped the images to contain just the area surrounding the food table, then ran the feature detector over that image. Figure 9 shows the cropped images of the video frame used in Figure 6. The yellow rectangles show the bounding boxes detected as birds. The top image shows birds detected by the LBP feature detector and the bottom the Haar feature detector. From these images, it can be seen that cropping the image reduced the number of false positives. Cropping the images also reduced the total number of frames without birds that had false positives, as seen in Table 3. Reducing the number of false‐positive detections resulted in an increase in precision using the cropped images, as seen in Table 2.

**Table 3:** For each feature detector, food table, and image size this table shows the number and percent of frames where a bird was detected with no bird present

Detector	Frames without birds and false positives	Percentage of frames without birds and false positives (%)	Frames with birds and true positives	Percentage of frames with birds and true positives (%)
table1B full image using Haar	8,927	81	1,116	61
table1B full image using LBP	6,408	58	936	51
table2B full image using Haar	8,314	75	358	49
table2B full image using LBP	6,682	60	253	34
table1B cropped image using Haar	8,835	79	1,093	60
table1B cropped image using LBP	5,316	48	915	50
table2B cropped image using Haar	8,222	74	335	46
table2B cropped image using LBP	5,193	47	241	33

Note

It also shows the percent and number of frames with true‐positive detections using an IoU threshold of 0.5 when birds were in the frame.

**Figure 10:**
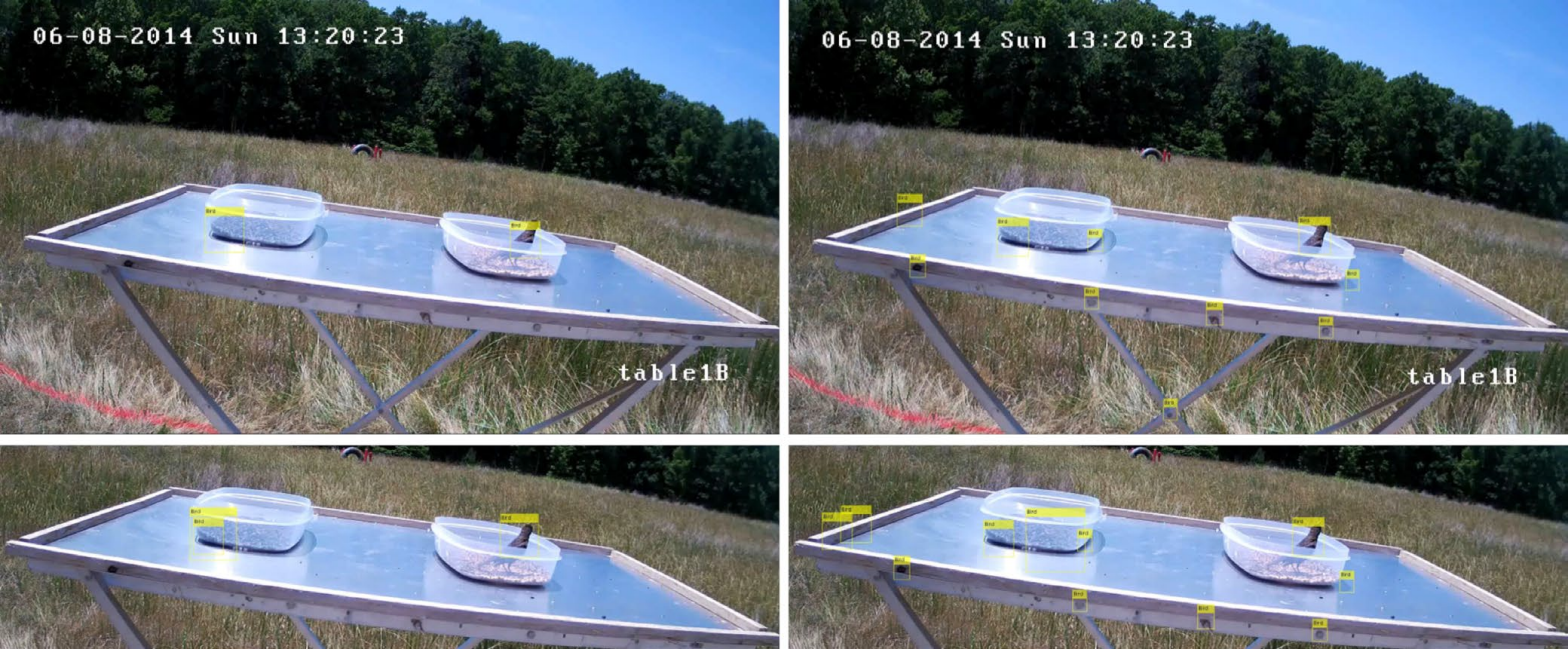
These images are the same video frame of table1B. For both the full and cropped images, the left side shows the detections using the LBP feature detector and right is the Haar feature detector. The yellow rectangles labeled “Bird” are the bounding boxes where birds were detected using the object detection algorithm. In each image, only the portion of the bird outside of the birdseed container is inside the bounding box. This resulted in an IoU less than the 0.5 threshold in each case

Table 3 also shows the number and percent of frames with a true‐positive detection when a bird was on the food table using an IoU threshold of 0.5. From Table 3, it can be seen that the LBP feature detector had fewer frames with true positives than the Haar feature detector. In Figure 6, it appears that both detectors correctly identify the bird in the frame. Using an IoU threshold of 0.5, only the Haar feature detector has a true‐positive detection. Figure 7 shows the zoomed in area around the bird seen in Figure 6 with the ground truth bounding box highlighted in green. The yellow rectangles are the bounding boxes for the bird detected using the LBP feature detector on the left and the Haar feature detector on the right. The IoU using the LBP feature detector of this bird is 0.46 and the IoU using the Haar feature detector is 0.52. The LBP feature detector identified only the body of the bird, while the Haar detector encompassed the entire bird. Due to the large variety of possible orientations of the birds, as seen in Figure 5, the detectors often identify the main body of the bird and exclude the feet and/or tail. The detectors omitting part of the body is also common when the birds are inside or behind the birdseed containers. Figure 10 shows the bird detections in a frame from table1B. The yellow rectangles are the bounding boxes where birds were detected using the object detection algorithm. The left images used the LBP feature detector and the right used the Haar feature detector. The top images are the full frame images, and the bottom images are cropped to include just the area around the food table. The partial obstruction from the birdseed container caused the detectors to identify just the area above the dish, while the human ground truth includes the body inside the dish. In both the full and cropped images, the Haar and LBP feature detectors identify only the portion of the bird outside of the birdseed container. None of these detections had an IoU over 0.5 and were therefore counted as false positives.

While the precision increased by cropping the images, the recall slightly decreased. This can be seen in Table 2 where the recall for each detector on the two food tables decreased on average by 0.012. The recall decreased because the number of true‐positive detections slightly decreased by cropping the images. Table 3 shows the number of frames with true‐positive detections using an IoU threshold of 0.5. Cropping the images shows a decrease in the number of frames with true positives which demonstrates the decrease in total true‐positive detections. The cropped images used the same detection algorithm as the full images. This algorithm was trained using the full images; thus, there were no images where the bird was at the top edge of the image. By reducing the amount of space between the birds and the edge of the image, the detection methods had a harder time correctly detecting the birds. This can be seen in Figure 9 where the bird's head is at the top edge of the cropped image. The LBP feature detected essentially the same bounding box in the cropped image as the full image, resulting in a IoU of 0.41. By cropping the image, the Haar feature detected a smaller bounding box, that of just the body of the bird excluding the tail, with an IoU of 0.49. Cropping the images reduced the recall of the system slightly because some of the birds were close to the edge of the photograph resulting in detection bounding boxes counted a false positives using an IoU of 0.5.

**Figure 11:**
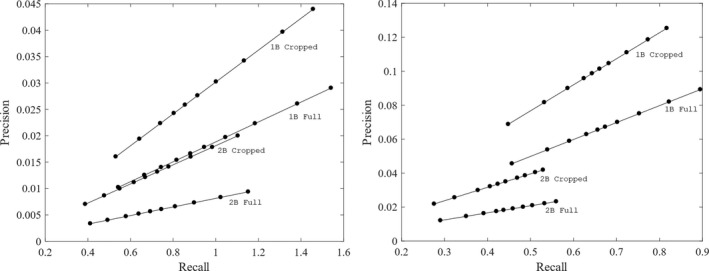
These plots show the precision versus recall for both detectors on the full and cropped images. The left plot used the Haar detector and the right plot is of the LBP detector. On each line, the filled circle closest to the origin is the IoU threshold of 0.5 and decrease by increments of 0.05 moving left to right. Each line is labeled with the food table and size of the image used. From these plots, it can be seen that the LBP feature detector had consistently higher precision values than the Haar feature detector. This is because the the LBP feature detector had fewer false positives. It can also be seen that the Haar feature detector had higher recall than the LBP feature detector, meaning that there were more true‐positive detections using the Haar feature detector. In all cases, the precision increased by cropping the images to include just the area around the food table. This is due to the reduction in false positives created by clouds and background clutter in the image. The recall decreased slightly by cropping the images. This is because the object detector was trained using the full images and therefore expected some amount of space between the bird and the edge of the image. Cropping the images decreased this space and resulted in fewer true‐positive detections of the birds near the edges of the images

The frequent false‐positive detections due to the object detector omitting parts of the birds led us to explore using smaller IoU thresholds. We found that by reducing the IoU threshold to 0.35 in all cases but table2B using the LBP detector the recall value increased to over 0.6 and the precision increased between 0.001 and 0.02. Figure 11 shows the precision‐recall plots using each detector type. The plot on the left is the Haar feature detector and the plot on the right is the LBP feature detector. Each line is labeled with the food table and size of the image. The dots represent the IoU thresholds. The dot closest to the origin is the IoU threshold of 0.5, and the IoU threshold decreases by increments of 0.05 from left to right. This figure shows that in all cases both the precision and recall increase by decreasing the IoU threshold. Note that the recall values are sometimes higher than 1 because the detector correctly identified the bird in more than one bounding box.

Figure 11 shows that the Haar feature detector has higher recall than the LBP feature detector for each food table and image size, but the LBP detector has higher precision. This is because the Haar feature detector more accurately detected the birds while the LBP feature detector had fewer false‐positive detections per frame. This figure also shows that reducing the false positives created by background clutter via cropping the images increased the precision for every detector on both food tables. There is a slight reduction in recall from cropping the images. This is because reducing the amount of space between the edge of the photograph and the birds reduced the number of true‐positive identifications by each detector.

## 4 DISCUSSION

Finite human attention span will always be a factor in visual analysis of video files, limiting the amount of data that can be collected and analyzed. Creating an automated detection system can reduce the amount of manpower needed, extend the feasible length of future experiments, allow for real‐time detection, and allow a single investigator to run multiple experiments simultaneously. For this study, we focused on the effectiveness of using a cascade object detector for automated bird detection to determine the presence of birds on food tables. We have shown that this technology can be used to detect bird presence, although improvements will need to occur before the system can be fully automated.

With an IoU threshold of 0.5, the Haar feature detector had a recall of over 0.5. This shows that the cascade object detector algorithm can be a useful tool for detecting bird presence in an image with a complex background. The main disadvantage to the current algorithm is the high number of false‐positive detections, which resulted in low precision values. The skew of the data toward having no birds in the frame, the low camera angle to the table, and lighting variations contributed to the high number of false positives, often resulting in shadows, reflections, and clouds being detected as birds. Cropping the images to just include the area around the food tables resulted in higher precision and lower recall in each case. The False Alarm Rate also decreased in each case, meaning that there were fewer false positives. These improvements are not sufficient to allow the system to be fully automated yet. Possible future improvements to decrease false‐positive detections include preprocessing the images, increasing the training set size, and combining features used in the algorithm. While this technology is not yet suited to replace human counting, combining automated detection with human observers could reduce time and the number of images that need to be examined manually. Possible schemes for this include setting a baseline of false‐positive detections reducing the total images a researcher needs to view, or using the automated detector to alert a researcher to take a second look at a specific area within an image. Reducing the amount of human analysis opens up many possibilities for further understanding of birds in natural environments.

This testing also provided us with some insights for designing future SonicNets experiments. On the positive side, the Audio Spotlight speaker, designed for indoor use, was functional after running for 33 days at 100% duty cycle for 8 hr a day in the humid Virginia summer. However, we also confirmed that more birds must be present to quantify the effectiveness of the acoustic deterrent. Designing and verifying the accuracy of the computer automated detection techniques gave us accurate counts of the birds visiting the food tables each day, but found no statistically significant impact due to the bird deterrent, because very few birds visited the food tables, and never in groups larger than 5. This field test had been designed to replicate earlier aviary experiments, but no flocks consistently visited either food table (Mahjoub et al., 2015). Nevertheless, we were able to show that an automated computer detection system can be used to detect birds in a field experiment not designed for computer vision.

## 5 CONCLUSION

The decreasing cost of cameras, image processing, and digital storage allows researchers to generate and store massive amounts of digital imagery. The time needed to manually analyze these images will always be a limiting factor for experimental design and analysis. This study demonstrates a technique for automated bird detection and counting in a field test without optimizing the test for computer vision. We have shown that cascade object detectors using LBP and Haar features can correctly identify birds in a complex environment. Without editing the images, the Haar feature detector had a recall value of 0.5 with an IoU threshold of 0.5, but improvements are necessary for the algorithms to be fully automated and replace human analysis. In its current state, combining this technique with human researchers could reduce the manpower needed to analyze similar data. Possible strategies for this include setting a baseline of false‐positive detections, to reduce the total number of images a researcher needs to view, or using the automated detector to alert a researcher to take a second look at specific area within an image. Reducing the amount of human analysis opens up many possibilities for further understanding of birds in natural environments.

One way this technology could further our understanding of birds is by using imagery of convenience created by bird feeder cameras to track migratory behavior. The introduction of products like the Nest Hello (https://store.google.com/us/product/nest%5C_hello%5C_doorbell?hl=en-US) doorbell cam allows people to create enormous amounts of digital imagery that they are often willing to share freely. These cameras are inexpensive, and it is a matter of time until like‐minded people start using them to record their bird feeders. Machine learning is also becoming more accessible to the general public, and a cascade object detector like the one used in this study would be straight‐forward to implement on this kind of imagery to detect the presence of birds at the feeders. Using this bird detection on connected cameras worldwide could give researchers real‐time information on bird distribution. Adding species labels, either by humans or improved automatic machine learning techniques, would increase our understanding of species migration. This technology could be used in coordination with programs like the Cornell Project FeederWatch (https://feederwatch.org/about/project-overview/) to create a robust amount of data not reliant solely on volunteer data collectors.

There are some simple steps that can be taken to make the images better suited for computer vision detecting birds. The first is to have a training set with more images of birds in the frame. The data used in this study were highly skewed toward images without birds, resulting in many false‐positive detections. Recording only when motion is detected would significantly increase the number of frames with birds, allowing the positive training set to be much larger. Another way to improve the images for computer vision would be to image the birds in a way that will reduce the orientations of the birds, background clutter, reflections, shadows, and obstructions. Since the images were intended to be analyzed by human investigators, the positioning of the camera and low camera angle to the food tables did not take any of these things into account, and created a more difficult imaging scheme. If the field of view of these cameras include areas where the birds are not likely to visit, the simple step of cropping the images can improve the accuracy of the automated detection. By cropping the images in this data, we increased the precision of the detector. All of these steps together could dramatically increase the ability of the algorithm the detect birds and could easily be done with the images of convenience shared using Wi‐Fi connected cameras.

The cost to acquire and store large amounts of video imagery continues to plummet. Crowdsourcing data could allow for data points throughout the world to better understand the distribution and migration of birds throughout the year. As technologies improve for automated analysis of images from field data, we can look forward to being able to easily re‐analyze imagery previously acquired for some other purpose and perhaps filed away in a drawer (Rosenthal, 1979).

## CONFLICT OF INTEREST

None declared.

## AUTHOR CONTRIBUTIONS

E. Simons collected and performed the data analysis, advised by M. Hinders. All authors contributed to the preparation of the manuscript.

## Data Availability

The data set will be archived via the William & Mary Applied Science Department web pages at https://www.as.wm.edu/Nondestructive.html.
